# Predictors of spousal coercive control and its association with intimate partner violence evidence from National Family Health Survey-4 (2015-2016) India

**DOI:** 10.1186/s12889-021-12232-3

**Published:** 2021-11-29

**Authors:** Suman Kanougiya, Muthusamy Sivakami, Saurabh Rai

**Affiliations:** School of Health Systems Studies (SHSS), Tata Institute of Social Sciences (TISS), Mumbai, India

**Keywords:** Coercive control, Controlling behaviour, Intimate partner violence, Violence against women, National Family Health Survey-4, India

## Abstract

**Background:**

The feminist theory posits that spousal coercive control is not random but a purposeful and systematic men’s strategy to control and dominate their female partners. The frequency of coercive control is more than emotional, physical, and sexual intimate partner violence (IPV). Coercive control is usually mistaken with psychological abuse when it is not and has recently gained independent attention within the spectrum of IPV. The role of socioeconomic factors in determining coercive control and associations between coercive control and form of IPV is less researched.

**Objective:**

We aimed to examine sociodemographic and socioeconomic predictors of spousal coercive control and its association with IPV (past 12-months).

**Methods:**

We analysed data of 66,013 ever-married women aged 15-49 from the National Family Health Survey (NFHS)-4 (2015-2016). Estimates involved bivariate and multivariate logistic regression models, and marginal effects prediction.

**Results:**

The prevalence of spousal coercive control is more commonly reported by 48% of women than the prevalence of IPV 25% (emotional 11%, physical 22%, and sexual 5%) in the past 12 months. Adjusted odds ratio indicate that women having three and more children (aOR 1.1, 95% CI: 1.0-1.2), women work status (1.1; 1.1-1.2), husband’s secondary (1.1; 1.1-1.2) or higher education (1.1; 1.1-1.2), and husband alcohol consumption (1.7; 1.6-1.7) increase the odds of coercive control. In the fully adjusted model coercive control independently increased the likelihood of experiencing emotional (aOR 2.8.; 95% CI: 2.6, 3.1), physical (2.2; 2.1, 2.3), and sexual (2.5; 2.3, 2.8) IPV in the past 12 months; and with an increase in each additional indicator of coercive control acts, the likelihood of physical, sexual, and emotional IPV further increases. When women reported six indicators of coercive control, the predicted proportion of women experiencing emotional 53%, physical 45%, and sexual IPV was 25% in the fully adjusted model.

**Conclusion:**

Coercive control limits women’s social support and contacts contributing to low self-esteem, self-efficacy, and poor mental health. The purpose of this study is to highlight that understudied coercive control is more common than other forms of IPV and is a potential risk factor for physical, sexual, and emotional IPV independently. The inclusion of coercive control in interventions is crucial to prevent form of IPV. Survivals long-term safety and independence can be secured if the current protection law against domestic violence is extended to encompass coercive control.

## Background

Nearly one in every three women experienced lifetime physical and/or sexual IPV globally, and some countries have a prevalence as high as 50% [1]. IPV contributes to poor reproductive, maternal and child health outcomes, injuries, suicide, homicide, and mental disorders [2]. Emotional, physical, sexual, and economic IPV is the manifestation of domination and control that needs to be addressed globally [1]. Some authors argue that one of the definitions of power is simply controlling a partner’s behaviour [3, 4]. Coercive control behaviour is defined as “making a person subordinate or dependent by isolating them from sources of support, exploiting their resources and capacities for personal gain, depriving them of the means needed for independence, resistance, and escape, and regulating their everyday lives” [5]. Coercive control is to make a person dependent by isolating a person from their family or friends, exploiting them, monitoring movements, restricting access to information and services, not allowing to work outside of the home and depriving them of independence, and regulating their everyday behaviour [6, 7]. Although IPV is defined as a “pattern of coercive control” [8], the measurement of IPV in research has mainly focused on violent and aggressive acts, such as physical and sexual violence and sometimes emotional abuse, and rarely on the coercive relationship. Stark (2007) describes controlling behaviour or coercive control as oppressive behaviour grounded in gender-based privilege. Coercive control sometimes is treated as a subset of psychological abuse. However, coercive control is based on the notion that one can and will punish another for non-compliance [9]. The opportunity for resistance exists but at a cost. Compliance with coercive control may work as a “reward” or as “buttering” to avoid the punishment that could be more severe and violent. Both victims and non-victims of coercive control experience other forms of IPV (physical, sexual and psychological). However, greater power to punish and greater likelihood of being punished is predicted to result in both greater compliance and greater resistance [10].

Coercive control is considered a critical predictive factor of physical and/or sexual IPV [11, 12]. Coercive control can precede, motivate, or increase the likelihood of other forms of violence in relationships, particularly when coercive control does not achieve the desired effect [11–14]. Coercive control predicts sexual, physical and emotional violence far better than prior assault [15, 16]. Physical, sexual, and emotional IPV are well predicted by demographic and socioeconomic characteristics [17]. Nevertheless, the association between coercive control and forms of IPV and the role of socioeconomic factors in determining coercive control is less researched within IPV than physical and sexual violence [7, 18, 19]. Women reporting one or more coercive control by their spouses varied from 21% in Japan [2], 30% in Malawi [20], 32.1% in rural Vietnam [21], 49% in Nepal [22], 63% in Nigeria [23], to around 90% in the United Republic of Tanzania [2]; suggesting male control over female behaviour is normative to different degrees in the various settings and differences in norms and survey methodolgy may have contributed to variations in prevalence. Polyvictimisation or multiple abuse victimisation at the same time among women by their husbands is high [24, 25]. Often coercive control and physical violence are those IPV that overlapped to the largest extent [26]. For example, nearly 40% of women in Peru who had ever suffered physical and/or sexual violence IPV, had also experienced at least four forms of coercive control, compared to 7% of women who had never experienced IPV. Another study conducted in Sweden reported that four out of ten women who experienced jealousy from their spouse were also exposed to physical and sexual violence [27]. Consistently across 15 sites of the WHO multi-country study settings, men who exhibited coercive control toward their partners also physically and/or sexually or both abused their female partners, indicating the significance of the partner’s number of coercive control was associated with a higher risk of physical or sexual violence, or both among women [7, 28, 29]. Coercive control in an abusive relationship escalated the risk of fatality by nine-folds [15], and cases involving coercive control were more likely to result in serious harm than cases involving discrete acts of physical violence [5, 30–32]. Victims of coercive control are at a higher risk of suffering from Common Mental Disorders (CMDs) and suicidal ideation [33, 34]. Coercive control also targets a victim’s autonomy, equality, liberty, social supports, and dignity in ways that compromise the capacity for independent, self-interested decision making vital to escape and effective resistance to violence [12].

In India, social and family structures are highly patriarchal and directly or/and indirectly promote and validate male dominance over women. Studies indicate that IPV is strongly associated with male patriarchal values, and women who experienced patriarchal control from their husbands were also more likely to experience physical and sexual IPV or sexual coercion [35–37]. India is a socially, culturally, and geographically diverse country, depicting considerable variation in IPV rate across its states ranging from 4% in Sikkim to 55% in Manipur [38]. Coercive control appears to be common in India, reported by 48% of ever-married women of reproductive age [38]. The rural-urban disparity in the experience of coercive control is stark. For example, around 43% of presently married women living in an urban residential area of Delhi experienced coercive control [39], whereas, in a rural tribal community in Rajasthan, 60% of women experienced it [34]. Another study carried out in urban informal settlements in Mumbai cited 71% of domestic coercive control [33]. Women in India experiencing coercive control by their partners were 3-8 times more likely to experience physical, sexual, or emotional IPV [33, 39, 40]. Coercive control was a strong predictors for CMDs and suicidal ideation [33, 34]. Studies from India have measured a range of socioeconomic characteristics as predictors of IPV [13, 41, 42], however, only a few studies have investigated the association between coercive control and IPV in India and rarely studies have focused on examining the role of various socioeconomic predictors of coercive control [33, 34, 39, 43, 44]. However, these studies represents a specific section of society. Thus, the role of socioeconomic factors in determining coercive control and its association with forms of IPV remains inconclusive. The role of coercive control in IPV and the socioeconomic predictors of coercive control need to be more thoroughly understood in the legal and intervention context to address social-cultural diversity, increasing our understanding of the etiology and consequences. By examining the socioeconomic predictors of coercive control, this study would inform efforts towards prevention and reduction of coercive control and IPV against women.

A study based on nationally representative data would provide more insights for a country like India with such large diversity. Therefore, the present study examines the role of socioeconomic variables in determining spousal coercive control and its association with physical, sexual, and emotional IPV among ever-married women aged 15-49 by using the recent data from the National Family Health Survey (NFHS)-4 (2015-2016).

## Data and method

### Settings

The Ministry of Health and Family Welfare (MoHFW), Government of India (GoI), initiated the NFHS surveys with International Institute of Population Sciences (IIPS) as a nodal agency to provide high-quality data on population and health indicators. The information provided by NFHS rounds assists policymakers and programme administrators in planning and implementing population, health, and nutrition programmes. The NFHS uses standardised questionnaires, sample designs, and field procedures to collect data on reproductive and child health, fertility, family planning, infant and child mortality, nutrition of women and children, the quality of family and health welfare services, socioeconomic conditions, and domestic violence to provide national and state-level estimates. The NFHS 2015-2016 is the fourth series of national data source covering 29 states, seven union territories, and 640 districts nationwide with a representative sample of households.

### Study design

NFHS-4 included a domestic violence module (DVM), administered in the sub-sample of households selected for the state module. NFHS-4 was carried out in two phases, from 20 January 2015 to 4 December 2016, following a stratified two-stage sample survey design conducted in urban and rural settings. Keeping the WHO ethical guidelines on violence against women (VAW) [45], only one eligible woman per household was randomly selected for the DVM. Before the interview, informed consent from each respondent was obtained. The best effort was to maintain privacy, and the domestic violence module was not implemented if privacy could not be obtained. The detailed methodology, with complete information on the survey design and data collection, has been published in the survey report [38].

### Sample size

In total, 699,686 eligible women age 15-49 with a response rate of 97% completed the interview. Special weights were applied to select one woman per household, ensuring the domestic violence subsample was nationally representative. In total, 83,397 women were selected for the domestic violence module, and only 4% could not be successfully interviewed due to privacy issues or other concerns. Overall, 79,729 women completed the domestic violence module. In the current study, women never in the union were excluded (*n*= 13,545 includes— Gauna^1^ was not performed *n*=171) because they do not live with their spouse or in-laws. Many questions on IPV and coercive control may not be relevant and applicable to women never in the union, therefore, contributing to missing values/data. Missing data reduces the statistical power of a study and often produce biased estimates, leading to invalid conclusions [46]. Hence, in total, 66,013 ever-married women aged 15-49 were included as the final sample.

### Variables

The domestic violence module of NFHS-4 obtained information from ever-married women aged 15-49 years of age whose current husband or most recent husband exhibits at least one of the following sets of behaviours or acts.

### Outcome variable—coercive control

Coercive control was assessed in the NFHS survey by using following six-questions without specifying any time frame. Women were requested to tell if these following apply to their relationship with their (last) husband— (a) is jealous or angry if she talks to other men; (b) frequently accuses her of being unfaithful; (c) does not permit her to meet her female friends; (d) tries to limit her contact with her family; (e) insists on knowing where she is at all times, and (f) does not trust her with any money. Furthermore, if exhibits, none of the above acts is coded as 0. We generated binary responses for each of the coercive control questions above and a final composite score of 0-6 describing the spouse’s intensity of coercive control analysis. Similar scales have been used in the WHO multi-country study [2].

### Exposure variable—intimate partner violence

#### Physical IPV

Physical IPV was referred to any exposure to one or several of the following acts against women by a current or former husband or partner in the past 12 months (a) push, shake, or throw something; (b) slap; (c) twist arm or pull hair; (d) punch with his fist or with something that could hurt; (e) kick, drag, or beat up; (f) try to choke or burn on purpose; (g) or threaten or attack with a knife, gun, or any other weapon.

#### Sexual IPV

Sexual IPV was referred to any exposure to one or several of the following acts against women by a current or former husband or partner in the past 12 months: (a) physically force his wife to have sexual intercourse with him even when the wife did not want to; (b) physically force his wife to perform any other sexual acts she did not want to; (c) force wife with threats or in other ways to perform sexual acts she did not want to.

#### Emotional IPV

Emotional IPV was referred to any exposure to one or several of the following acts against women by a current or former husband or partner in the past 12 months: (a) say or do something to humiliate wife in front of others; (b) threaten to hurt or harm wife or someone close to her; (c) insult wife or make a wife feel bad about herself.

If women answered “often” and “sometimes” to any set of the physical, sexual, and emotional violence questions, committed by a current or by a most recent husband in the 12 months preceding the survey, it was coded as (1) ‘Yes, experienced violence’, for each type of IPV separately. Women response to “never” or “yes, but not in the past 12 months” to all of these questions was coded as (0) ‘No, not experienced violence’, for each type of IPV. The internal consistency of physical, sexual, and emotional violence questions was assessed by computing the Cronbach’s Alpha. The results show overall good reliability [47] of emotional violence (α .73), physical violence (α .80), sexual violence (α .77), and coercive control (α .73).

### Sociodemographic variables

The analysis included demographic and socioeconomic variables as confounders based on previous studies [23, 39, 48]. Variables included for women—marital status (currently married, Widowed/Separated/Divorced); age (categorised as 15–24, 25–34, and 35-49); women’s education (no education, primary, secondary, and higher); the number of living children (0, 1, 2, and 3+); women’s occupation (not working and working currently). Spouse characteristics – husband’s schooling (no education, primary, secondary, and higher); husband’s occupation (not working, non-agricultural, agricultural, and skilled & unskilled manual); husband’s habit of consuming alcohol (yes or no). Place of residence (urban and rural); caste (schedule-caste, schedule-tribe, other backward castes (OBC) and general caste^2^ ); religion (Hindu, Muslim, and other religion); wealth index (Poorest, Poorer, Middle, Richer, Richest) corresponding to wealth quintiles ranging from the lowest to the highest.

### Statistical analysis

We tabulated frequencies and proportions of sociodemographic variables, responses to coercive control, physical, sexual, and emotional IPV. We examined the association between sociodemographic variables and coercive control by performing univariate and multivariate logistic regression. Further, we examined the impact of coercive control on the experience of the past 12 months of physical, sexual, and emotional IPV in a series of univariate and multivariate logistic regression models. We computed unadjusted and two adjusted models: the first model (aOR_1_) was adjusted for sociodemographic variables —respondent age, education, number of living children, religion, caste, asset quintile, residence, respondent and husband employment, and husband alcohol use. The second model (aOR_2_) was adjusted for sociodemographic variables and forms of IPV.

Additionally, we examined the effect of increasing numbers of coercive control on IPV forms in the past 12 months. We adjusted the logistic regression models in the same way as above (aOR_2_). For example, the model included coercive control as the exposure variable and physical IPV in the past 12 months as the outcome variable and was adjusted for sociodemographic and socioeconomic variables plus the other two forms of IPV (sexual and emotional IPV). A similar analysis was repeated for each form of IPV separately.

And then we predicted marginal effects and modelled the log-odds of the form of IPV as a step function from 0 to 1 act of coercive control, followed by a linear increase from 1 to 6 acts. We tested for non-linearity by fitting a quadratic term for the increase from 1 to 6. All estimates accounted for sampling weights, and analyses were performed in STATA 15.0 (StataCorp LLC).

### Enhancing the quality of data and safety of respondents

NFHS followed the recommendations made by Lori Heise and Mary Ellsberg, CHANGE and the WHO multi-country study of women's health and domestic violence, core protocol. Field staff received additional training in administering the domestic module using the rapport building and safety procedures established by the survey, including dealing with crises and preparing themselves emotionally for the work. Only one woman per household should receive the domestic violence module. The introductory sentence in the violence module included an additional informed consent procedure. Respondents were reassured about the confidentiality of the information. A participant’s information sheet (PIS) was distributed to the appropriate language across states and union territories. The PIS listed options and services that were available for women experiencing domestic violence and legal help and available services. PIS also contained an address where women in need could get information on domestic violence. The PIS was small enough to be easily hidden. More details are available at https://dhsprogram.com/Methodology/Survey-Types/DHS.cfm.

## Results

**Fig. 1:**
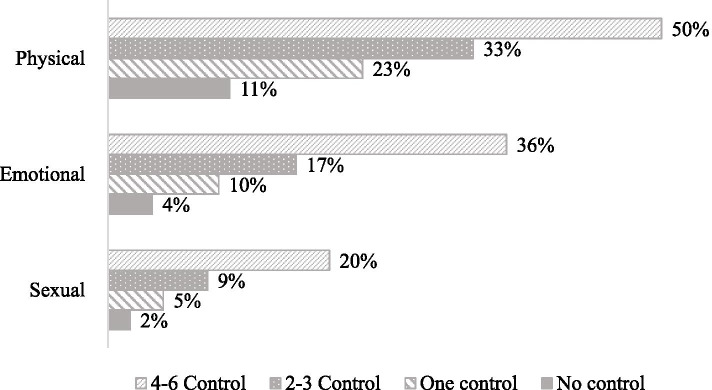
Proportion of Women Who Experienced Emotional, Physical, and Sexual Intimate Partner Violence in Past 12 Months by Number of Coercive Control Acts

Table 1 summarises characteristics, the experience of coercive control, and forms of IPV of 66,013 ever-married women. Most of the women were currently married, 95%. Around 9% had no children, 34% had had no schooling. A quarter of women (25%) were in remunerated work, 96% of women’s partner was in remunerated work, and 32% consumed alcohol. More than one-third of women identified themselves as Hindu, 21% of the general caste, and 71% of women were living in rural areas. Nearly 48% of women reported at least one type of coercive control by their spouses. The past 12 months IPV was 25% (physical 22%, emotional 11%, and sexual 5%). Furthermore, Figure 1 shows the percent of women reporting past 12 months forms of IPV with no, one, 2-3, and 4-6 number of acts of coercive control. The percent of women reported IPV was higher with number of acts of coercive control— Sexual (2, 5, 9, and 20%), emotional (4, 10, 17 and 36%), and physical IPV (11, 23, 33, and 50%).

**Table 1:** Sociodemographic profile, experience of spousal coercive control, and forms of intimate partner violence for 66, 013 ever-married women aged 15-49 years, India, 2015–16

Demographic profile	N	(%)
**Marital status**		
Currently married	62716	(95)
Widowed/Separated/Divorced	3297	(5)
**Age (Years)**		
15–24	10489	(16)
25–34	27568	(42)
35–49	27956	(42)
**Number of living children**		
0	6136	(9)
1	12610	(19)
2	22842	(35)
3+	24425	(37)
**Education**		
No education	22028	(34)
Primary	9669	(14)
Secondary	28187	(43)
Higher	6129	(9)
**Women employment**		
Not working	49355	(75)
Currently working	16658	(25)
**Husband education**		
No education	12776	(19)
Primary	9854	(15)
Secondary	34597	(53)
Higher	8579	(13)
**Husband’s occupation**		
Not employed	2674	(4)
Non-agriculture	20849	(32)
Agriculture	22363	(34)
Skilled and unskilled manual	19399	(30)
**Husband consume alcohol/drug**	20891	(32)
**Place of residence**		
Urban	19469	(29)
Rural	46544	(71)
**Caste**		
Scheduled caste	11686	(19)
Scheduled tribe	12108	(19)
Other backward castes	25574	(41)
General caste	13449	(21)
**Religion**		
Hindu	49546	(75)
Muslim	8614	(13)
Other religion	7814	(12)
**Wealth index**		
Poorest	12838	(20)
Poorer	13992	(21)
Middle	13790	(21)
Richer	13142	(20)
Richest	12251	(19)
**Any coercive control by spouse**	**31448**	**(48)**
Husband/partner jealous if respondent talks with other men	16320	(25)
Husband/partner does not trust the respondent with money	15307	(23)
Husband/partner insists on knowing where the respondent is	12971	(20)
Husband/partner does not permit the respondent to meet female friends	12776	(19)
Husband/partner tries to limit the respondent’s contact with the family	9457	(14)
Husband/partner accuses respondent of unfaithfulness	5268	(8)
**Any intimate partner violence (emotional, physical, sexual) in past 12 months**	**16674**	**(25)**
Emotional IPV	6944	(11)
Physical IPV	14158	(22)
Sexual IPV	3581	(5)
**All**	**66013**	**(100)**

Table 2 summarises the experience of coercive control by the socioeconomic profile of women and their effect on coercive control. The experience of coercive control did not differ by marital status. The proportion of women aged 15-24, women having three or more children, women with no schooling, currently working, women belonging to scheduled caste, having Hindu faith, from the poorest wealth quintile, and living in rural areas reported more coercive control by their spouses. Women whose husbands had no education, no employment, and consumed alcohol exhibited more coercive control than husbands without these characteristics. The multivariate logistic regression results show that characteristics like women having three and more children (aOR_1_ 1.1, 95% CI: 1.0-1.2), women’s working status (1.1; 1.1-1.2) were more likely to experience coercive control. Husbands who were having secondary education (1.1; 1.1-1.2), or higher education (1.2; 1.1-1.3), and a history of alcohol consumption (1.7; 1.6-1.7) were more likely to exhibit coercive control than the husband in the reference categories. On the other hand, with an increase in women’s age, education, and wealth quantile, and women belonging to the other religion, scheduled tribe, and general caste were less likely to experience coercive control than women in the reference categories.

**Table 2:** Association of sociodemographic variables with spousal coercive control for 66, 013 ever-married women aged 15–49 years India, 2015–16

	Coercive control					
	No		Yes			
	N	(%)	N	(%)	OR [95% CI]	aOR_1_ [95% CI]
**Marital status**						
Currently married	32860	(52)	29856	(48)	1	1
Widowed/Separated/Divorced	1705	(52)	1592	(48)	1.0 [1.0, 1.1]	1.0 [0.9 , 1.0]
**Age (years)**						
15–24	5032	(48)	5457	(52)	1	1
25–34	14258	(52)	13310	(48)	0.9 [0.8, 0.9]	0.8 [0.8 , 0.9]
35–49	15275	(55)	12681	(45)	0.8 [0.7, 0.8]	0.7 [0.6 , 0.7]
**Number of living children**						
0	3081	(50)	3055	(50)	1	1
1	7023	(56)	5587	(44)	0.8 [0.8, 0.9]	0.9 [0.8 , 0.9]
2	12587	(55)	10255	(45)	0.8 [0.8, 0.9]	0.9 [0.9, 1.0]
3+	11874	(49)	12551	(51)	1.1 [1.0, 1.1]	1.1 [1.0, 1.2]
**Educational**						
No education	10044	(46)	11984	(54)	1	1
Primary	4966	(51)	4703	(49)	0.8 [0.8, 0.8]	0.9 [0.8 , 0.9]
Secondary	15785	(56)	12402	(44)	0.7 [0.6, 0.7]	0.8 [0.8 , 0.8]
Higher	3770	(62)	2359	(39)	0.5 [0.5, 0.6]	0.7 [0.6 , 0.8]
**Women’s employment**						
Not working	26368	(53)	22987	(47)	1	1
Currently working	8197	(49)	8461	(51)	1.2 [1.1, 1.2]	1.1 [1.1, 1.2]
**Husband educational**						
No education	5935	(46)	6841	(54)	1	1
Primary	4919	(50)	4935	(50)	0.9 [0.8, 0.9]	1.0 [0.9, 1.0]
Secondary	18593	(54)	16004	(46)	0.7 [0.7, 0.8]	1.0 [1.0, 1.1]
Higher	5015	(59)	3564	(42)	0.6 [0.6, 0.7]	1.1 [1.1, 1.2]
**Husband’s occupation**						
Not employed	1304	(49)	1370	(51)	1	1
Non-agriculture	11716	(56)	9133	(44)	0.7 [0.7, 0.8]	0.9 [0.8 , 1.0]
Agriculture	11387	(51)	10976	(49)	0.9 [0.8, 1.0]	0.9 [0.8 , 0.9]
Skilled and unskilled manual	9789	(51)	9610	(49)	0.9 [0.9, 1.0]	0.9 [0.8 , 1.0]
**Husband consume alcohol**						
No	25508	(57)	19614	(43)	1	1
Yes	9057	(43)	11834	(57)	1.7 [1.6, 1.8]	1.7 [1.6 , 1.7]
**Caste**						
Scheduled caste	5430	(47)	6256	(53)	1	1
Scheduled tribe	6896	(57)	5212	(43)	0.7 [0.6, 0.7]	0.7 [0.6, 0.7]
Other backward caste	12548	(49)	13026	(51)	0.9 [0.9, 0.9]	1.0 [1.0, 1.1]
General caste	7724	(57)	5725	(43)	0.6 [0.6, 0.7]	0.9 [0.8, 0.9]
**Religion**						
Hindu	24903	(50)	24643	(50)	1	1
Muslim	4733	(55)	3881	(45)	0.8 [0.8, 0.9]	1.0 [1.0 , 1.1]
Other religion	4905	(63)	2909	(37)	0.6 [0.6, 0.6]	0.7 [0.7 , 0.8]
**Wealth index**						
Poorest	5201	(41)	7637	(60)	1	1
Poorer	6954	(50)	7038	(50)	0.7 [0.7, 0.7]	0.7 [0.7 , 0.8]
Middle	7405	(54)	6385	(46)	0.6 [0.6, 0.6]	0.7 [0.6 , 0.7]
Richer	7549	(57)	5593	(43)	0.5 [0.5, 0.5]	0.6 [0.6 , 0.6]
Richest	7456	(61)	4795	(39)	0.4 [0.4, 0.5]	0.6 [0.5 , 0.6]
**Place of residence**						
Urban	11027	(57)	8442	(43)	1	1
Rural	23538	(51)	23006	(49)	1.3 [1.2, 1.3]	1.0 [1.0 , 1.0]

*OR* crude odds ratio for coercive control, *aOR* adjusted odds ratio for coercive control, including all covariates in the table

Table 3 shows associations of coercive control with emotional, physical, and sexual IPV. The chi-square test of association results shows that women who experience coercive control were more likely to experience emotional (82% compared with 42%), physical (72% compared with 41%), and sexual (84% compared with 46%) than women who did not experience coercive control in the past 12 months. The results from univariate and multivariate logistic regression indicate that the experience of coercive control independently increased the likelihood of experiencing emotional (aOR_2_ 2.8.; 95% CI 2.6, 3.1), physical (2.2; 2.1, 2.3), and sexual (2.5; 2.3, 2.8) IPV in the last 12 months.

**Table 3:** Association of spousal coercive control with forms of intimate partner violence in past 12 months for 66, 013 ever-married women aged 15–49 years, India, 2015–16

	Coercive control						
	N	(%)	N	(%)	OR [95% CI]	aOR_1_ [95% CI]	aOR_2_ [95% CI]
**Emotional IPV**							
No	33277	(56)	25792	(44)	1	1	1
Yes	1288	(19)	5656	(82)	5.7 [5.3 , 6.0]	4.8 [4.5 , 5.1]	2.8 [2.6 , 3.1]
**Physical IPV**							
No	30661	(59)	21194	(41)	1	1	1
Yes	3904	(28)	10254	(72)	3.8 [3.6 , 4.0]	3.2 [3.0 , 3.3]	2.2 [2.1 , 2.3]
**Sexual IPV**							
No	33981	(54)	28451	(46)	1	1	1
Yes	584	(16)	2997	(84)	6.1 [5.6 , 6.7]	4.9 [4.5 , 5.4]	2.5 [2.3 , 2.8]

*OR* crude odds ratio, *aOR1* odds ratio adjusted with covariates for respondent marital status, age, number of children, education, religion, caste, socioeconomic quintile, respondent and husband education, employment, occupation, and husband alcohol use, *aOR2* odds ratio adjusted as aOR1 plus covariates for emotional, physical, and sexual violence

**Fig. 2:**
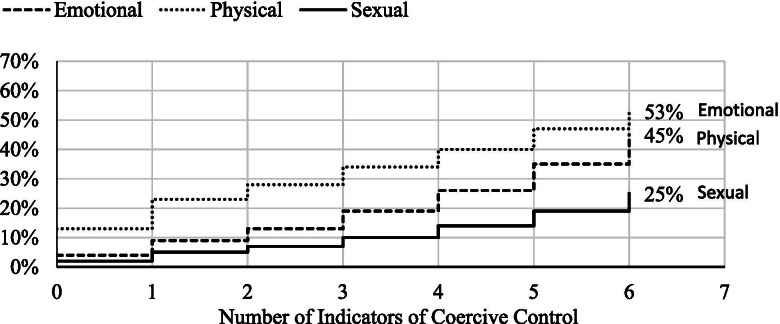
Risk of Intimate Partner Violence in Past 12 Months, Conditional on the Experience of 0-6 Acts of Coercive Control (adjusted for sociodemographic variables). aOR: odds ratio adjusted with covariates for respondent marital status, age, number of children, education, religion, caste, socioeconomic quintile, respondent and husband education, employment, occupation, and husband alcohol us plus covariates for emotional, physical, and sexual violence

Figure 2 presents the findings from conditional logistic regression models for the impact of coercive control on emotional, physical, and sexual IPV. For each outcome, predicted marginal effects are presented for the model adjusted with sociodemographic covariates. In the absence of coercive control, the predicted proportion of women with emotional violence was 4%, physical violence 13% and sexual violence 2%. The percent of women experiencing forms of IPV increased with an increase in each additional indicators of coercive control. When women reported six acts of coercive control, the predicted proportion of emotional IPV was 45%, physical 53%, and 25% sexual IPV in the past 12-months in the fully adjusted model.

## Discussion

In a sample of 66,013 ever-married women aged 15-49, spousal coercive control was more common than emotional, physical, or sexual IPV. Furthermore, the experience of emotional, physical, or sexual IPV was more among women who experienced one or more acts of coercive control than women who did not. Coercive control independently increased the risk of emotional, physical, and sexual IPV by 2-3 folds. Furthermore, the risk of experiencing emotional, physical, and sexual IPV increased each number of coercive control acts. The working status of women and secondary or higher education of husbands and husbands’ alcohol consumption increased the risk of experiencing coercive control. On the other hand increase in women’s age, education, and wealth quantile, the risk of coercive control decreased.

Our study’s prevalence of coercive control is in the range with studies conducted in various countries ranging between 21 to 90% by women in a heterosexual relationship [7, 11, 20, 23, 49]. The acts of coercive control, such as restriction, isolation, and control as abusive tactics, have also been experienced by women in other settings [7, 50, 51]. In our study, spousal coercive control was more common than emotional, physical, or sexual violence and can be equally or more threatening than emotional, physical, or sexual IPV, similar to other studies’ findings [52, 53].

Our study shows that younger women, women who had no education, working women, and women from the lowest wealth quantile are at a higher risk of experiencing coercive control. Socioeconomic characteristics such as age, education, working status of women, number of children, caste, religion, poor economic status of households, and husband consuming alcohol are strong predictors of IPV in India and other countries [7, 13, 23, 38, 42, 43, 54]. Results in our study show that working women are at a higher risk of reporting coercive control than non-working women. Working status is an indicator of women empowerment and her socioeconomic status in society, which is an important determinant of her husband’s behaviour. Literature suggests that a rise in women’s empowerment status threatens men’s dominant status, making women susceptible to IPV, including being controlled [13, 39, 55–57]. Our study shows that low husband education is associated with greater coercive control in the univariate analysis but lower coercive control in the multivariate regression. This could be because husbands with some level of education are aware of abusive acts which are criminalised and which are not. Hence, selectively make a strategy to establish dominance and control women as suggested by the feminist ideology that coercive control is a systematic strategy. However, the finding is opposite to the finding of another study conducted in India, where the adjusted odds ratio indicate that husbands who had 12 and above years of education are less likely to exhibit coercive control [39]. Studies carried out using the National Health Survey in Nigeria and Myanmar show that husbands’ education is not associated with women’s experience of coercive control [23, 48]. So far, limited studies have examined the role of socioeconomic characteristics on coercive control. Hence, more insights from other studies in the future are needed for a better understanding. Findings from our study show that husband’s alcohol consumption increased the risk of coercive control by 2-fold. Such association is consistent with findings from countries like Uganda, Zimbabwe, Vietnam, Argentina, Costa Rica, Kazakhstan, Nigeria, Sri Lanka and the ten countries in the WHO multi-country study [7, 21, 58].

Our study shows that spousal coercive control is a significant risk factor that independently triggers the likelihood of an experience of physical, sexual, or emotional IPV. This finding is consistent with other studies where strong positive associations (between 2 and 5 times) have been found between coercive control and risk of IPV [11, 59–61]. Our study also shows that with each increase in indicator of coercive control, the proportion of emotional, physical, or sexual IPV increases. The risk for violence directly increased with the number of coercive controls on the husband’s part across the diverse cultures studied [48, 62]. The link between male IPV and various socially coercive controls has been found cross-culturally [63] and in our study too. Using coercive measures to establish control and domination by Indian husbands in our study aligns with a feminist perspective of IPV. The feminist perspective explains how patriarchal societies normalize the idea of male domination to strengthen and validate a male-dominated social order and family systems. This male domination permeates relationships too, which permits men to exercise power and control over women in several ways, including violence [64, 65]. Our study focuses on the associations between coercive control and forms of IPV. Results provide empirical evidence supporting that coercive control is independently a strong risk factor and broadens our understanding of the genesis and consequences of male-to-female emotional, physical, and sexual violence.

Interventionists need to pay closer attention to socioeconomic factors while planning prevention design to reduce coercive control. For example, introducing microfinancing or income generation activities among women might further escalate the risk of coercive control or economic abuse. Likewise, higher education of husbands might make them strategically cleverer to introduce coercive control acts in the relationship, realising the difficulty victims would face to produce evidence against such abusive behaviour. The findings are important in planning and allocating efforts towards protecting, preventing, and reducing IPV against women. Coercive control reduces women’s social support by limiting their social contact with neighbours, friends, and family members and contributes to low self-esteem, self-efficacy, and poor mental health [34, 66, 67]. Evidence shows that besides the Protection of Women from Domestic Violence Act (PWDVA)-2005, the rate of help-seeking remains very low in India—nine in every ten women did not seek any help, and a little more than one percent has sought help from formal source, although women preferred informal sources such as a friend, neighbour, and own family than formal sources [38]. PWDVA-2005 defines physical, sexual, emotional/verbal, and economic abuse; however, coercive control is yet to be recognised in the remedial framework. Perpetrators realise that such controlling acts would not be taken seriously [68] under the law and continue to use controlling tactics effectively with or without violence to limit social support and access to women’s resource.

Our study addresses several knowledge gaps. First, the study provides evidence that coercive control is independently a risk factor to physical, sexual, or emotional IPV in India. Second, the present study provides evidence that coercive control does not occur in isolation, but the other forms of IPV are also present with coercive control. Third, an increase in coercive control acts further escalates the risk of physical, sexual, or emotional IPV. Coercive control may be considered as an early sign of other forms of IPV as suggested by feminist ideology. Intervention in the initial state of coercive control might work as a buffering factor to prevent physical, sexual, and emotional IPV.

## Limitations

Due to the nature of the cross-sectional study, we could not draw a cause and effect relationship. We could not control for the impact of other potential risk factors, such as disability of the respondents and their husbands, and the mental health status of respondents, husbands, and other family members in multivariate logistic regression models [46].

## Conclusion and policy implications

Spousal coercive control is more common than emotional, physical, and sexual IPV in India, affecting almost half of ever-married women. Coercive control is a strong predictor for other forms of IPV and limits women’s day to day opportunities and social support, contributing to low self-esteem, self-efficacy, and poor mental health. Previous studies show that high husband education was negatively associated with physical, sexual, and emotional IPV. However, studies have shown mixed effects of husband education on coercive control. It is necessary to consider the socioeconomic profile of the perpetrator at the intervention level. Intervention in the initial state of coercive control might work as a buffering factor to prevent physical, sexual, and emotional IPV. With an increase in each indicator of coercive control, the risk of experiencing IPV increases. Not in totality, but each coercive control act needs necessary attention at intervention and prevention programmes. IPV survivors' long-term safety and independence can be secured if current protections against domestic violence are extended to encompass coercive control. Careful intervention strategies around coercive control would provide multiple benefits, such as mitigating coercive control, preventing IPV, and reducing poor mental health outcomes for women.

## Data Availability

All the data published so far are available for download through The Demographic Health Survey (DHS) Program's data distribution system. The DHS website belongs to ICF International. For more details on obtaining and downloading NFHS data, please visit: www.DHSprogram.com

## Footnotes

1 *Gauna* is a ceremony associated with consummation of marriage in certain parts of India. In communities that follow this practice, the marriage is only consummated after the *Gauna* is performed.

2 General caste/General Class/General Category/Open Category/Forward Caste is a term used in India to denote Caste groups that have been historically privileged due to the belief that these castes are superior in the social hierarchy. Thus, the members of these caste groups are on average ahead of members from other caste socially and often economically too.
